# Acceptability of a hypothetical preventative HIV vaccine among people who use drugs in Vancouver, Canada

**DOI:** 10.1186/s12889-020-09202-6

**Published:** 2020-07-09

**Authors:** Taylor Fleming, Jenna Valleriani, Cara Ng, Lisa Maher, Will Small, Ryan McNeil

**Affiliations:** 1 British Columbia Centre on Substance Use, Vancouver, BC Canada; 2 Interdisciplinary Graduate Studies Program, University of British Columbia, Vancouver, BC Canada; 3 National Institute for Cannabis Health and Education, Toronto, ON Canada; 4 The Kirby Institute for Infection and Immunity, Faculty of Medicine, University of New South Wales, Sydney, NSW Australia; 5 The Burnet Institute, Melbourne, VIC Australia; 6 Faculty of Health Sciences, Simon Fraser University, Burnaby, BC Canada; 7 Department of Medicine, Yale School of Medicine, New Haven, CT USA; 8 Program in Addiction Medicine, Yale School of Medicine, Yale University, 367 Cedar Street, New Haven, CT USA

**Keywords:** Vaccines, HIV/AIDS, HIV prevention, Qualitative research

## Abstract

**Background:**

As research on HIV vaccines continues to advance, studies exploring the feasibility of this intervention are necessary to inform uptake and dissemination strategies with key populations, including people who use drugs (PWUD).

**Methods:**

We conducted 25 in-depth qualitative interviews examining HIV vaccine acceptability among PWUD in Vancouver, Canada. Participants were recruited from an ongoing prospective cohort of HIV-negative PWUD. Data were coded using NVivo, and analyzed thematically.

**Results:**

Acceptability was framed by practical considerations such as cost and side effects, and was influenced by broader trust of government bodies and health care professionals. While an HIV vaccine was perceived as an important prevention tool, willingness to be vaccinated was low. Results suggest that future vaccine implementation must consider how to minimize the burden an HIV vaccine may place on PWUD. Centering the role of health care providers in information dissemination and delivery may assist with uptake.

**Conclusions:**

Our findings suggest improvements in care and improved patient-provider relationships would increase the acceptability of a potential HIV vaccine among this population.

## Background

As candidate prophylactic HIV vaccines progress through clinical trials [1–3], it is critical to assess acceptability among potential recipients to inform implementation strategies. Past studies have found that the acceptability of HIV vaccines depends on a variety of important factors, including perceptions of safety, potential side effects, distrust of health care systems and providers, and considerations of social risks (e.g., discrimination) [4, 5]. These works have generally focused on populations believed to be at high risk of HIV acquisition, such as sexually active youth [6, 7], people who use or inject drugs [8, 9], sex workers [10, 11] and men who have sex with men (MSM) [12, 13]. Uptake among these key populations presents an important challenge for the dissemination of new biomedical prevention efforts, including people who use drugs (PWUD). Further, how PWUD perceive and engage with prevention interventions can be influenced by their experiences of co-morbid conditions (e.g., hepatitis C, mental illness) [14, 15], as well as by less visible social, structural, and economic barriers [16–19]. Previous research has found willingness to receive an HIV vaccine among PWUD was generally low, and shaped by perceptions of self-efficacy [8] and stigmatization of HIV [20]. This is of particular importance when contrasted with research examining acceptability and uptake of a hepatitis B (HBV) and C (HCV) vaccines, for which reports of hypothetical and real-world uptake are attributed to lack of knowledge of HBV and HCV [9, 21, 22], rather than stigma.

Much previous work on the acceptability of HIV vaccines among PWUD has relied predominantly on quantitative research [23–25] and has failed to consider the complex interplay of social, structural and environmental contexts and their impact on health outcomes in this population. The broader literature increasingly identifies the need to shift away from a focus on individual choices and behaviours to understanding of underlying conditions and contexts, in order to address HIV risk [19, 26]. For example, gendered power relations may play a role in women’s HIV-related vulnerability [27, 28], and considerations of contextual factors such as vulnerability to sexual and physical violence, distinctively shape women’s HIV-risk profiles [29, 30].

As research on candidate HIV vaccines continues to advance, centering the voices of PWUD as a key population for biomedical prevention interventions will allow for a more nuanced examination of uptake and feasibility currently lacking in available studies. In this qualitative study, we explore the perceptions of PWUD towards a hypothetical HIV vaccine in Vancouver, Canada, and their implications for the acceptability and uptake of an efficacious vaccine.

## Methods

This ethno-epidemiological study draws on semi-structured interviews conducted with 25 participants of the Vancouver Drug Users Study (V-DUS). V-DUS is a prospective cohort study of HIV-negative people who use drugs, operating in Vancouver, Canada since 1996. This cohort has been described in detail elsewhere [31]. In short, V-DUS participants are recruited through snowball sampling and street outreach, and are eligible to participate in the cohort study if they are 18 years or older, HIV negative, and used illicit drugs in the past 30 days at baseline. Participants complete a standardized interviewer-administered questionnaire and provide blood samples at baseline and bi-annual follow-up visits. Ethno-epidemiology aims to merge both epidemiological and qualitative methods to increase understanding of how social-structural contexts impact patterns of health and social harms [32].

V-DUS participants have well-established relationships with our research program through their involvement in the longitudinal cohort study. V-DUS participants who were eligible for the present study were contacted by a V-DUS team member, and invited to participate in a one-time interview at a storefront research office located in Vancouver’s Downtown Eastside neighbourhood—a low-income inner city neighbourhood with a high prevalence of HIV and illicit drug use. Participants were eligible for this study if they had completed a follow-up interview with V-DUS in the previous six months, were current regular users of illicit drugs, and were HIV-negative. Participant characteristics are reported in Table 1. We aimed to oversample women and Indigenous persons relative to their representation among the local drug-using population in recognition of the ways they are uniquely impacted by HIV risk. The study was approved by the research ethics board of Providence Health Care and the University of British Columbia.

**Table 1:** Participant characteristics

	Participants (***n*** = 25)
**Age**	
*Mean*	50.2
*Range*	34–73
**Gender**	
*Men*	14 (56%)
*Women*	11 (44%)
**Ethnicity**	
*White*	10 (40%)
*Indigenous*	13 (52%)
*Other*	2 (8%)
**Drugs use in past 30 days**^**a**^	
*Heroin*	15 (60%)
*Cocaine (powder)*	7 (28%)
*Crack cocaine*	4 (16%)
*Crystal methamphetamine*	8 (32%)
*Fentanyl*	10 (40%)
**Frequency of drug use**	
*Daily*	15 (60%)
*3–4 times per week*	3 (12%)
*One or fewer times per week*	7 (28%)
**Would receive HIV vaccine**^**b**^	
*Yes*	12 (48%)
*No*	10 (40%)
*Undecided*	2 (8%)

^a^Note participants could select multiple substances

^b^Hypothetical approved preventative HIV vaccine

Semi-structured interviews with 25 participants took place in a private room, and were conducted by JV and CN using an interview guide to facilitate discussion. Both interviewers are extensively trained in qualitative research methods, including interviewing, and have prior experience conducting interviews with PWUD. Before beginning the interview, JV and CN explained the study, reviewed the consent form with participants, and received written informed consent. Interviews focused on general immunization history, perceptions of HIV risk, current risk behaviours, knowledge of public health efforts and campaigns around HIV, and willingness to receive a hypothetical preventative HIV vaccine. For the purposes of this study, this vaccine was assumed to be an intramuscular injection [33, 34]. The interview guide developed for this study (see Additional file 1) focused on a hypothetical proven HIV vaccine, however, lines of questioning were flexible enough to allow for more general discussion of HIV vaccines, including clinical trial participation. Interviews were audio-recorded, and lasted between 45 and 75 min each. Participants received $30 cash honorarium for their time. Upon reaching saturation, interviews were then transcribed verbatim, coded, and analyzed thematically using NVivo 12. Participants were assigned pseudonyms using a random name generator.

We drew on both deductive and inductive approaches [35] during analysis to focus on the uptake and acceptability of a hypothetical preventative HIV vaccine, potential concerns around a hypothetical vaccine, relationships with health care service providers, and practical considerations that shape acceptability (e.g., cost, dosing regimens, side effects). We developed an initial coding framework informed by a priori categories extracted from the interview guide, and our research team (JV, CN and RM) met after reviewing 3–5 transcripts to further refine the coding framework based on emerging themes. Once final themes were established, data were recoded to ensure trustworthiness of final themes. Two team members (JV and CN) independently coded each of the remaining transcripts to establish inter-coder reliability, and the research team met regularly to discuss findings as a group.

## Results

### HIV vaccine acceptability

#### Willingness to receive an HIV vaccine

Participants were generally knowledgeable about and accepting of vaccines other than the influenza vaccine, with which many reported they had prior negative experiences. Participants viewed vaccines as an important public and individual health tool, and most had received them as necessary (e.g., childhood vaccinations). Few participants were unaware of what a vaccine is or how it works, although these individuals were more skeptical about a hypothetical preventative HIV vaccine. Further, participants acknowledged the important role that vaccines played in both public and individual health, although they were unwilling to receive any vaccine that was not demonstrated to be effective and accepted among the general public. For example, while most participants, including ‘Charlotte’ (49-year-old white woman) considered a future HIV vaccine useful, many reported not wanting to be among the first to be vaccinated, whether as a participant in a clinical trial of a candidate vaccine or as part of new public health initiative for a vaccine shown to be efficacious:

*I don’t want to be one of the guinea pigs though … I don’t want to be, like, you know, what I was saying, they have to do trials and stuff? I don’t want to be in that group of people that do the trials. I don’t care how much money they give me.*

Similar to others, Charlotte invoked the image of “*guinea pigs*” to describe clinical trial participants, and would under no circumstances participate as a test subject to demonstrate efficacy of a candidate vaccine.

Conversely, other participants believed a preventative HIV vaccine would be useful, but would be unwilling to receive such a vaccine in any capacity, regardless of demonstrated efficacy. These participants framed their unwillingness to participate through their perceived risk to HIV exposure. Similar to other participants, ‘Andy’ (62-year-old white man) characterized his level of risk as low, by contrasting himself to other PWUD who may participate in conventionally high HIV-risk behaviours (e.g., sex work, MSM), or who participate in the same behaviours as himself but in ‘riskier’ ways (e.g., higher frequency of injecting):

*Well if you’re MSM or you’re an injection drug user, I think it would be a smart thing to take if it was proven that it was working. If you were sure, you know 99 percent that it was going to prevent it, then you should, especially if you are engaging in things that we know, there are two ways of getting HIV, let’s face it – sex and injection drugs – that’s it. So, if you are engaging in those risky behaviours, then why not. You would be stupid not to because the alternative is really shitty … I don’t have any, you know, sex no, there is no need* [for a vaccine]*, and I don’t share rigs. The one time when I do my occasional shot now I use a brand-new rig out of a package.*

Within these narratives, participants sought to distance themselves from stigma associated with drug use, while simultaneously reinforcing stigmatization of PWUD who may be among the most socially and economically marginalized.

Were a vaccine proven to be efficacious through clinical trials and offered as part of a new public health initiative, participants still viewed the vaccine to be somewhat experimental, with Charlotte continuing “*I would want to see other people get the vaccine first before trying it,”* even though, as members of a key population PWUD may be among the first offered a hypothetical preventative HIV vaccine. Many felt it was important to wait before getting a newly approved vaccine as part of a public health initiative because of any potential unknown consequences, and multiple participants expressed concern about being one of the first to get a new vaccine. The idea of a new preventative HIV vaccine concerned participants, who were unsure if that would come with unknown and potentially long-term side effects, and whether safety of the vaccine had been established; this is despite the implication that such a vaccine would have already been approved for public use, suggesting that public acceptance of a vaccine’s efficacy shaped willingness to receive a vaccine just as much, if not more than clinical acceptance (e.g., clinical trial results):

*I mean, it would have to be like really, really tested, and really show that it works, you know? Like what proof do you know? The only proof is would you take it and then you go and have sex with an HIV-positive person and see if it works or not.* (‘Simon,’ 44-year-old white man)

Moreover, for Simon and others, the evidence supporting an HIV vaccine still had to be considered with the risk of it not working as intended when deciding whether or not to vaccinate. For example, ‘Nick’ (58-year-old Black man) questioned the role of public support in relation to the seasonal influenza vaccine, noting that despite public health campaigns purporting the benefits of vaccination, many who were vaccinated still became ill with the flu each year: “*I’m taking it* [flu vaccine] *because they* [public health messaging] *tell me it’s good for me. I’m hoping they’re telling me the truth, but I don’t know.*”

All participants expressed that they would want more information about the effectiveness of an HIV vaccine and its side effects before they exposed themselves to it. For most participants, mild to moderate side effects would be acceptable, such as a sore arm or fever, and would not impede uptake, as Nick explained:

*I’d have to hear them* [the side effects] *… I don’t want to, as I said, I don’t want to go blind … I’d have to hear what the side effects are, and do I really want to go through it. Like, if the side effects are just headaches, I could take that.*

#### Institutional trust and mistrust

Skepticism towards a hypothetical HIV vaccine was predominantly framed by a mistrust in government and pharmaceutical companies, as well as by past negative experiences within health care systems. Many participants felt that these agencies and institutions cared little about the public, generally, and PWUD, in particular. These perceptions were shaped by negative personal experiences with government agencies (e.g., criminal justice system, child welfare system), as well as reports from others within their peer networks. ‘Harvey’ (60-year-old white man) explained:

*You can’t always trust the government. I think they think they’re making the best decision in your interest. I don’t know, at my age, I know things about the government that they’ve done to friends of mine and at the time they were doing these things they weren’t so forthcoming with what was really going on.*

Some participants spoke about rejecting future vaccination campaigns because of a long-standing suspicion of government initiatives. For example, ‘Laura’ (39-year-old Indigenous woman) explained, *“The government’s looking out for themselves. The people are nothing”* when discussing her faith in government-sponsored vaccination initiatives. When not relating this mistrust to past negative experiences with governmental initiatives and institutions, participants cited conspiracy theories, such as population control or concerns about the “*chemical composition*” of vaccines, as why they would be skeptical of any future preventative HIV vaccines, regardless of proven efficacy. For ‘Luke’ (62-year-old white man), targeting a high-risk group, such as PWUD, under the guise of HIV vaccination was viewed as a potential strategy to eliminate socially and morally stigmatized groups:

*Who are they gonna get rid of first? They’re gonna get rid of the drug addicts, the prisoners … you know, because we’re overpopulated … it’s common sense. The planet can only hold so many people, and there’s only so much food, there’s only so much water, there’s only so much of everything. And we keep going the way we’re going, what’s gonna happen, right? I mean, who goes first, right? It’s you or I?*

Participants who subscribed to conspiracy theories surrounding vaccinations had no personal experiences to support these claims. Rather, belief in these theories originated from reports through peer networks and personal research: “*I just read that* [conspiracy] *on the internet and I got scared*” (‘Marco,’ 38-year-old white man).

While participants were generally distrustful of government and pharmaceutical companies, the majority spoke extensively about the trust they had in their individual health care providers with regards to medical care and information about vaccinations, suggesting that participants trusted clinicians to scrutinize public health policy and potentially harmful government initiatives for them. Participants unanimously said they would want to receive information about the vaccination from a health care professional, such as a doctor or nurse, because they considered them knowledgeable and trusted them. The vast majority of participants had a regular physician, and the relationships these participants had developed with their health care providers helped shape their attitudes towards a potential HIV vaccine, as Nick described:

*I’d need to sit down and talk to my doctor and hear what they got to tell me about it. I just can’t jump in to it because someone says we’ve got a vaccine. I want to know about the vaccine. I want to know if it can help me. I want to know if it can hurt me. I want to know about it before I take it … I’ve got a really good doctor.*

Luke echoed this trust in his individual physician as a source of information about an HIV vaccine.

*If my next-door neighbours tell it to me, well, you know what I mean? I might believe it, I might not, and then I don’t know if I, you know. But if my doctor told me to, I’d take it as gospel, basically, right?*

Some participants indicated that they would defer to their doctor’s authority or medical expertise, and were open to future vaccinations even though they did not believe they were at high risk for HIV. For example, ‘Donald’ (34-year-old Indigenous man) said, “*If they* [my doctor] *really thought I needed it, I probably would do it.”* Here we see that doctors carry authority with these participants in ways which could increase the acceptability and uptake of a preventative HIV vaccine. Even those participants who stated they would not be vaccinated (see Table 1) said that they would consider it if their doctor felt it was very important.

### HIV vaccine feasibility

#### Compulsory vaccination

As an extension of this more general distrust for government, most participants rejected the idea of a compulsory HIV vaccination policy for adults. Some participants raised concerns that targeting certain key populations, such as PWUD, would be discriminatory or further stigmatizing, while most asserted their individual autonomy to make health care decisions for themselves or in conjunction with their physician, as ‘Michelle’ (50-year-old Indigenous woman) highlighted: *“It’s a little invasive and intrusive … I just think that people should be able to make their own decisions.”* It should be noted that while health care providers seem to play a key role in participants’ decision to participate in new HIV prevention technologies, participants were strongly opposed to any type of coercive or compulsory approach to vaccination.

Half of our participants were, however, open to the idea of a tested and approved HIV vaccine being delivered to children in schools, even as a mandatory vaccination, and particularly before young people become sexually active. Some of this was related to concerns about the stigma of accessing an HIV vaccine, which participants believed would be eliminated if it were given to everybody uniformly at a young age as part of a childhood immunization schedule, in contrast to being targeted at high-risk individuals and populations.

#### Immunization schedule and cost

Immunization scheduling was also an important consideration in uptake and feasibility. While in general fewer doses were more acceptable, most participants indicated a preference for a single injection with no follow-up ‘booster shot.’ Approximately half reported that they would accept more doses on a schedule if required. However, many, including ‘Andy’ (62-year-old white man) cited the challenges of adhering to an immunization schedule, particularly a longer immunization schedule, while actively using drugs:

*Well that’s an issue with drug users, especially with drug users, because getting them to come in for follow ups are hard, and if the vaccine is based on the fact that you have to have three of them, say you had to have it three times two months apart, that could be an issue … but you never know with a drug user where, they could be in jail, they could be in a fucking different province, they could be anywhere. And so the follow up would be difficult.*

More broadly, Andy underscores the barriers to engaging in preventative HIV care for criminalized populations within the context of drug prohibition, as the act of meeting daily survival needs and the risk of law enforcement lends a level of unpredictability to participants’ lives, which may prohibit regular follow-up care.

In general, participants reported that they would want to receive the vaccination at a fixed site clinic, and very few individuals preferred receiving vaccinations through outreach services (e.g., ‘door to door’ or mobile campaigns). Such services may have implications for privacy, confidentiality, and social stigmatization, as Nick acknowledged:

*I wouldn’t want to do it. I don’t want everybody knowing my business... if you’re going to this place, then everybody thinks you got AIDS … I go to my own doctor, no one knows what I got*.

Participants identified cost as a major barrier or deterrent to vaccine uptake. Participants unanimously agreed that a preventative HIV vaccine should be free for vulnerable populations and those on limited incomes. Within this, some believed that a tested and approved HIV vaccine should be free of charge because they viewed it as a critical public health issue. Michelle was among the few participants who would be potentially willing to pay for a preventative HIV vaccine depending on the cost ($20–$100):

*Because we are fucking Canadians and we’re not from the USA… There should be no reason why we shouldn’t have free anything … especially vaccines in regard to health and stuff. No matter what risk group we’re in, we deserve it …Doesn’t matter what do we do in our life, what we do on the fucking sidelines in our home and it’s our business.*

The above quotation is notable in light of the focus on behavioural risk factors and the transmission of HIV. Michelle highlights that individuals hold autonomy over their lives, even if these choices place them at risk for HIV acquisition. Michelle also draws on the right to access (free) health care within the context of Canada’s publicly funded health care system, including new vaccinations, asserting that cost should not be a barrier to uptake. This also demonstrates the importance of limiting the financial burden vaccinations may place on individuals.

## Discussion

HIV vaccine acceptability among PWUD in our study was framed by practical considerations such as concerns about cost, vaccine schedules, and side effects. Acceptability was also influenced by perceptions and trust of government bodies, for-profit pharmaceutical industries, and health care professionals. In our study, a HIV vaccine was generally considered a useful preventative tool by PWUD, although willingness to personally receive such a vaccination was low.

While roughly half of our participants were willing to receive a hypothetical preventative HIV vaccine, willingness to participate amongst these individuals was near-unanimously conditional on widespread uptake and acceptance of this vaccine as safe and effective by the general public, and most rejected compulsory HIV immunization for adults. Despite this, many suggested a mandatory HIV vaccination policy for children, framing it as a way to protect future generations and achieve high coverage because of its potential to eliminate the stigma associated with being identified in a high-risk group later in life. Previous studies, however, have suggested a mandatory policy would be unlikely, as it requires endorsement and acceptance by the general population [36, 37]. The development of strategies for HIV vaccination uptake specifically tailored to PWUD, especially those considered at high risk or hard-to-reach, would help to ensure broad HIV vaccine coverage, particularly considering those who are at higher risk of transmission report being less likely to receive a preventative HIV vaccine [25]. While past hepatitis B research has found that, for PWUD willing to receive a preventative vaccination, cash incentives can increase the likelihood of uptake and completion of a vaccine schedule [22, 38], implementation of an HIV vaccine will require the development of strategies to specifically target initial vaccine uptake, particularly given the stigmatization of HIV and reluctance to engage with any HIV-associated services. Moreover, it is important to consider how traditional public health campaigns for HIV prevention have targeted particular high-risk groups [39], which may contribute to the stigmatization of those who are already marginalized. New approaches to HIV prevention must consider how stigma can affect or impede the uptake of new interventions.

Our findings also suggest that the patient-provider relationship is a critical component to reaching PWUD and that improvements in care, as well as patient-provider relationships, would increase the acceptability and uptake of a potentially new HIV vaccine among PWUD. This reported trust in health care providers was somewhat surprising considering the consistent lack of trust in government agencies and institutions shaped by negative past experiences within the health care system reported by our participants. Further, past research with PWUD has described prevailing attitudes of mistrust of health care providers and authorities as a major barrier to optimal engagement in preventative care, including vaccine uptake [21, 40]. Our participants’ narratives demonstrate that doctors with established positive relationships with PWUD patients exert considerable influence on individuals’ decision to consider being vaccinated against HIV, and that this group is critical to engagement in preventative care for such key populations. This is consistent with other studies that report that positive relationships between PWUD and health care providers lead to better health outcomes [41, 42]. This is also consistent with previous childhood vaccination research demonstrating that trust in health care providers is a key factor in parents’ decisions to vaccinate their children, and that this relationship is an important site of intervention in increasing vaccination uptake [43, 44]. While doctors may influence decisions by PWUD to be vaccinated, most of our participants described a conversation with their doctor as part of their decision making around uptake of a licensed HIV vaccine. Strategies to encourage uptake of a potential HIV vaccine by PWUD must also consider social and structural barriers which shape access to, and experiences with, health services and government bodies. In particular, the mistrust in government reported by our participants has significant implications for vaccination campaigns, including skepticism around candidate and licensed hypothetical vaccines, resulting in lower uptake and feasibility of these products. Broad institutional mistrust (e.g., government conspiracies, “Big Pharma”) has also been implicated in vaccine hesitancy within the contexts of childhood vaccinations [45] and communicable disease pandemics such as the 2009 H1N1 event [46]. Decoupling public health policy and practice from pharmaceutical companies and political powers will be an important step in promoting uptake of a potential HIV vaccine, particularly for PWUD, whose lived realities are criminalized by these very institutions.

To encourage real-world uptake of a future preventative HIV vaccine among PWUD, barriers such as cost, dosing regimens and mode of dissemination will need to be addressed. However, the impact of stigma as a barrier to uptake continues to underpin HIV prevention efforts, as is consistent with past research on intervention implementation among PWUD [47, 48]. For example, many of our participants did not want to discuss HIV-related topics (e.g., health services, risk behaviours) in detail, and when speaking to vaccination administration, indicated that were they to receive it, they would want to receive this vaccination in a clinic because of concerns around privacy, and not wanting to utilize HIV-specific agencies or services for fear of being identified as someone with HIV. This suggests that public health interventions should consider encouraging more positive HIV vaccine attitudes, and the creation of partnerships with health care providers can help offer accurate and destigmatizing information around HIV risk and risks of a potential vaccine as important piece of disseminating an HIV vaccine.

While this study adds to the small body of qualitative research on HIV vaccine acceptability, and highlights how social and structural barriers may shape access to this intervention for PWUD, this study has some important limitations. The V-DUS cohort is made up of both former and current PWUD, and is generally an older cohort of individuals [49] which may limit the generalizability of the findings to younger PWUD. Further, despite being overrepresented within our sample, Indigenous participants’ perspectives on HIV vaccine acceptability did not speak to racialized experiences, meaning that when asked directly by the interviewer, they did not feel being indigenous impacted their risk of HIV transmission, or access to vaccines, including information and conversations from their health care provider. Additionally, participants generally reported that that being Indigenous did not impact their access to other support and health services, nor did they bring up the impact of being Indigenous on health care access or vaccine access independently. Given that Indigenous peoples in Canada experiences inequitable access to health care, and a disproportionate burden of risk of HIV, future vaccine acceptability research should examine how race and histories of colonization impact these views. Lastly, the current context, the Downtown Eastside of Vancouver, Canada, is unique in that it represents a greater concentration of harm reduction and health services and PWUD than likely found elsewhere, and as such, a majority of our sample had a primary health care physician. Considering the importance of the patient-provider relationship in acceptability and uptake of an emerging HIV vaccine, populations of PWUD less engaged with health care services may be more difficult to reach in dissemination.

## Conclusion

While PWUD in the current study demonstrate a general acceptance of vaccines, they were less willing to receive a hypothetical preventative HIV vaccine when considering the potential for unknown long-term risks, were one to be proven effective and made publicly available. The lack of willingness by our participants to be vaccinated was also related to a distrust of government bodies and pharmaceutical companies, often juxtaposed against a reported trust in their health care providers. This highlights the importance of consolidating engagement of PWUD with health care providers as new prevention technology, such as HIV vaccines, emerge. However, even despite willingness to discuss this with a health care provider, PWUD did underscore the importance of agency in health care decision-making, rather than a compulsory policy for adults. Centralizing the role of health care providers in the dissemination of information and delivery of the HIV vaccine may assist with uptake among PWUD. Practically, vaccine schedule, cost, and where the vaccine would be administered (due to privacy concerns), can also shape uptake and dissemination. Thus, in the future, strategies should be tailored to meet the needs of PWUD and other high-risk groups to promote broad HIV vaccine coverage.

## Supplementary information

**Additional file 1.** Interview guide

## Data Availability

The data underlying these findings is presented in this manuscript in support of presented themes and subthemes as anonymized participant quotations. Further data cannot be made publicly available as per study approval by the research ethics board of Providence Health Care and the University of British Columbia. Interested researchers may direct data access requests to the corresponding author.

## Abbreviations

HIV: Human immunodeficiency virus
MSM: Men who have sex with men
PWUD: People who use drugs
V-DUS: Vancouver Drug Users Study
