# Supplementary material for: Acceptability of a hypothetical preventative HIV vaccine among people who use drugs in Vancouver, Canada
Source: BMC Public Health. 2020 Jul 9;20:1081. doi: 10.1186/s12889-020-09202-6 (PMC7350753; doi:10.1186/s12889-020-09202-6)
Supplement: Supplementary file 1 — Additional file 1. Interview guide [file 12889_2020_9202_MOESM1_ESM.pdf]

AN ETHNO-EPIDEMIOLOGICAL STUDY OF PERCEPTIONS OF THE ACCEPTABILITY AND FEASABILITY OF AN HIV VACCINE AMONG PEOPLE WHO USE DRUGS

QUALITATIVE INTERVIEW COVER SHEET

Interviewer:____

Date and Time:____

Cohort Code: ____

Filename: ____

DEMOGRAPHICS – DO NOT TURN ON AUDIO RECORDER

1. **What do you identify your gender as? (Check as many as apply)**
   - ☐ Woman
   - ☐ Man
   - ☐ Other____________________

2. **How old are you?**

   ____

3. **What do you identify your race or ethnicity as?**
   - ☐ White (Caucasian)
   - ☐ Indigenous / Aboriginal
   - ☐ East or Southeast Asian
   - ☐ South Asian
   - ☐ Black / African-Canadian
   - ☐ Other__________________

4. **What do you identify as your sexual orientation? (check all that apply)**
   - ☐ Straight
   - ☐ Gay
   - ☐ Lesbian
   - ☐ Bisexual
   - ☐ Two-spirited
   - ☐ Other:____________________

5. **What type of housing are you currently living in?**
   - ☐ House
   - ☐ Apartment
   - ☐ SRO Hotel (privately-owned)
   - ☐ SRO Hotel (publicly-owned)
   - ☐ Shelter
   - ☐ Friend’s place
   - ☐ Unsheltered / Outside
   - ☐ Other:______________________________________

6. **Are you currently living with any of the following?**
   - ☐ HIV/AIDS
   - ☐ Hepatitis C
   - ☐ Diabetes
   - ☐ COPD
   - ☐ Mental illness
   - ☐ Other:____________________

7. **Which of the following substances have you used in your lifetime?**
   - ☐ Cocaine (powder)
   - ☐ Crack cocaine (rock)
   - ☐ Crystal Methamphetamine
   - ☐ Heroin
   - ☐ Fentanyl
   - ☐ Opioids (extra-medical)
   - ☐ Marijuana
   - ☐ Alcohol
   - ☐ Other:____________________

8. **Which of the following substances have you used in the past <u>30</u> days? (Check all that apply and <u>Circle</u> drug of choice)**
   - ☐ Cocaine (powder)
   - ☐ Crack cocaine (rock)
   - ☐ Crystal Methamphetamine
   - ☐ Heroin
   - ☐ Fentanyl
   - ☐ Opioids (extra-medical)
   - ☐ Marijuana
   - ☐ Alcohol
   - ☐ Other:____________________

9. **How often are you currently using your <u>drug of choice</u>?**

☐ Daily ☐ 3-4 times per week ☐ One or fewer times per week

10. How often are you currently using <u>other drugs</u>?

☐ Daily ☐ 3-4 times per week ☐ One or fewer times per week

11. Which methods have you used to consume drugs in the past 30 days? (Check all that apply)

☐ Inject ☐ Smoke / Inhale ☐ Snort ☐ Ingest / Swallow

12. Which of the following ways have you made money in the <u>past 30 days</u>? (Check all that apply)

☐ Full-time employment ☐ Part-time employment ☐ Drug selling
☐ Sex work ☐ Recycling / binning ☐ Panhandling
☐ Social assistance ☐ Other:______________________

AN ETHNO-EPIDEMIOLOGICAL STUDY OF PERCEPTIONS OF THE ACCEPTABILITY AND FEASABILITY OF AN HIV VACCINE AMONG PEOPLE WHO USE DRUGS

QUALITATIVE INTERVIEW GUIDE

Interviewer:____

Date and Time:____

Filename: ____

DIRECTIONS

Thank you for agreeing to share your experiences as a person who uses drugs regarding your perspectives on a potential HIV vaccine. As we discussed during the consent process, today you will be taking part in an approximately 30-60-minute interview about your experiences in with drug use, sexual behaviour, and vaccinations, among other things. Please note that this interview will be audio recorded. Before we begin, please remember that everything that you share with us is confidential, and your information or identify will not be shared with anyone unless required by law. Also, please keep in mind that there are no right or wrong answers to these questions, and you do not have to answer any questions that you are not comfortable answering.

IN-DEPTH INTERVIEW – START RECORDING

I. CURRENT LIFE SITUATION	
**SOCIAL & STRUCTURAL ENVIRONMENT**	
Where have you been spending most of your time over the past month?	• *Where do you stay most days?*<br>• *What do you do there?*
Who have you been spending time with over the past month?	• *What friends, family or other people have you been seeing/mainly hanging out with?*<br>• *Tell me about these relationships?*<br>• *What kinds of things do you do with the people you spend time with?*
Do you feel you can access harm reduction materials, if you want to?	• *Why or why not?*<br>• *Where do you get your harm reduction materials?*<br>• *Is there anything that would make it easier for you to access harm reduction supplies?*
Have you had any recent interactions with the police?	• *Have you had a negative experience from using drugs in a public setting from the police?*<br>• *Do the police in any way influence your drug use? If so, how?*<br>• *Do the police in any way influence your day to day life? If so, how?*<br>• *Have you encountered more or less police since the overdose crisis became public?*<br>• *Have your interactions with the police changed at all since the overdose crisis became public?*

	• *Do you think the police response to the overdose crisis has been good or bad?*
**INCOME GENERATION**	
What do you currently do to make money?<br><br>***Note: Ask targeted follow-up questions on the specific types of formal/informal income generation engaged in.***	• *Do you supplement this in other ways?*<br>• *How long have you been making money this way?*<br>• *Can you describe what a typical day is like for you when making money?*<br>• *Does your drug use impact how you make money?*<br>• *Have the ways in which you've made money changed at all because of the recent overdose crisis? Could you describe to me any of these changes?*
Could you tell me what are the advantages and disadvantages of the ways you make money?	• *How is it better than other ways?*<br>• *How is it worse than other ways?*
**DRUG USE**	
How would you describe your drug use in the past month?	• *Which drugs do you use? How often?*<br>• *How long have you been using these drugs?*<br>• *Of the drugs you use, which is your drug of choice? How often do you use it?*<br>• *If you can't find your drug of choice, do you use another? Which drug?*<br>• *Where do you use drugs?*
Can you walk me through the process of a typical time that you might use drugs in the last month?	• *Do you have a routine you follow? What's your routine?*<br>• *What are you taking and how (smoking, ingesting, injecting, a combination)?*<br>• *Is there a clean surface needed, and if so, is one available to you?*<br>• *How about other supplies, are they available? For instance, clean needles/pipes, a washroom etc.*<br>• *(If applicable) Do you clean your skin before injecting?*<br>• *Would you describe it as a safe environment?*<br>• *Are people usually present?*<br>• *Do you do anything to limit your risk of overdose? What?*
Where do you get your drug use materials from?	• *Do you find you can easily get needle/pipes/etc. when you need them?*
(If applicable) Do you inject yourself?	• *If not, who does it for you?*<br>• *Do you share injecting equipment with your partner?*
Do you ever need assistance injecting?	• *(If so) How do you find assistance? Who helps you?*<br>• *How does this impact where and when you inject drugs?*
Do you ever use drugs in public spaces?	• *Can you describe these settings? (e.g. an alley, public bathroom)*<br>• *Would you describe this as a safe environment? Why?*

	• *Would you describe this as a clean environment? Why?*<br>• *Why would you or would you not use in public?*
**GENDER**	
Are you in a relationship?	• *For example, are you married? Have a boyfriend or girlfriend?*<br>• *Do you have children? If so, are they in your custody? Who has custody of them?*<br>• *Does your partner do drugs? If so, do you share drugs with your partner? Do they share with you?*<br>• *Do you and your partner use drugs together?*
(**If applicable**) Do you inject yourself?	• *If not, who does it for you? Does this impact when and where you inject?*<br>• *Do you share injecting equipment with your partner?*
**(If applicable**) Do you use your own pipe to smoke?	• *Where do you get your pipes from?*<br>• *Are pipes easy to access for you?*<br>• *Do you and your partner share pipes? Do you share with other people also?*
**INDIGENOUS LIVED EXPERIENCE (IF APPLICABLE)**	
Do you think that being Indigenous impacts your drug use?	• *Why or why not?*<br>• *As an Indigenous person, are there barriers or obstacles to using overdose prevention or safe consumption sites? Can you explain what these might be?*<br>• *As an Indigenous person, are there barriers to receiving treatment or support services? Can you explain what these might be?*<br>• *Is there anything that would make it easier to access treatment or support services?*
When accessing treatment or support services, do you think you're treated differently by staff or other people using the service?	• *In what ways?*<br>• *Can you describe an experience or provide an example?*
Do you ever feel unsafe when accessing treatment or support services?	• *In what ways?*<br>• *Can you describe an experience or provide an example?*
(**If applicable**) Do you feel that people of Indigenous ancestry have impacted differently than other groups by HIV?	• *How so? Why?*<br>• *How do you feel about these impacts? For example, do you ever feel that they are unfair?*<br>• *Do you think that existing treatments and supports are relevant to Indigenous peoples? Why or why not?*<br>• *Do you feel Indigenous people have specific needs that should be better incorporated into treatment and prevention services? Why or why not? Can you give some examples?*

	• *Do you feel Indigenous voices are being included in treatment and prevention services?*
II. PERCEPTIONS OF RISK AND RISK-REDUCTION STRATEGIES	
Can you tell me about the sorts of activities that you view as being “risky” or “less risky” with regards to HIV transmission?	• *Do you feel like you are ever at risk for HIV? Tell me about why.*<br>• *When you think about your own risk for getting HIV, what kinds of sexual behaviour do you feel might be “risky”? Why do you feel some behaviour could be more risky than others?*<br>• *When you think about your own risk for getting HIV, what kinds of drug-related behaviour do you feel could put you at risk for HIV?*<br>• *Do you ever engage in injection-related practices that you feel might put you at risk? Tell me about that.*
**(If reported syringe-sharing)** Can you tell me about the last time that you borrowed a syringe or lent one to someone else?	• *What were the circumstances surrounding syringe-sharing? For example, where were you injecting?*<br>• *Who did you share your syringe with and what is your relationship like with that person? For example, were they an intimate partner or someone you share drugs with?*<br>• *Were the circumstances surrounding syringe-sharing different than your usual injecting patterns? If so, how?*<br>• *Have you previously shared syringes? If so, how were these situations the same and different?*
**(If recently initiated injecting)** Can you describe to me the circumstances around when you started injecting?	• *For example, were you with anyone else when you first injected drugs? If so, did they play a role in your initiation into injecting?*<br>• *Did anyone influence your transition to injecting? If so, how did they influence this?*<br>• *What was your relationship like with this person? For example, how do you know them?*<br>• *Could you tell me about injecting for the first time? How did you prepare the drugs?*<br>• *How did you learn to inject? For example, did someone else help you to inject for the first time?*
**(If reporting sex without a condom)** Can you tell me about the circumstances when you would have sex without a condom?	• *What was your relationship like with the person you were having sex with? For example, were you with a regular partner or someone new?*<br>• *Broadly speaking, why did you not use a condom? For example, did you not have one with you? Did you not feel safe using one?*<br>• *Have you had sex without a condom previously? How were these time different or similar?*
Now I would like to discuss some of the strategies you use in your day-to-day life to prevent getting HIV. Could you tell me about the kinds of things you do (or have done in the past) to prevent yourself from getting HIV?	• *What HIV prevention strategies do you feel are important for people who use drugs to help them prevent getting HIV?*<br>• *For example, what kinds of **services** do you feel are available to help you manage various HIV prevention strategies?*

	• *How do you feel about HIV risk-reduction services such as Insite and other safe consumption sites?*<br>• *Do you ever access any of these services to help with injecting practices? Is this an important service for you? Tell me about that.*<br>• *Are there different sorts of practices that you engage in to prevent HIV acquisition? Tell me about those.*<br>• *Do you feel that people who use drugs continue to be at risk for HIV despite the availability of some programs and services that are available? Why?*
**The negotiation of sexual transmission of HIV:** *We are also interested in how you negotiate HIV status with your sex partners. Is this a discussion that you think is important to have? Tell me about that.*	• *How do you initiate conversations about HIV and sex?*<br>• *Some of our participants have previously told us that it's harder to talk about sex and HIV, depending on if you are a guy or a girl. What are your thoughts on talking about condoms and the risk of HIV for you as someone who identifies as a [insert gender identity here]?*<br>• *How do you think talking about condoms and sex would be different for someone who is of a different gender? Why?*
**The negotiation of injection-related transmission of HIV:** Have you ever discussed HIV with someone you were injecting drugs with and sharing a syringe?	• *(**If yes**) How did that conversation occur? Was this an important conversion for you to have? Why?*<br>• *(**If no**) What this something you would have liked to talk about? Tell me about that.*
We are also interested in how HIV risk-related behaviour changes over time. For example, in our previous work, people have told us that they found their experiences with both sexual and injection-related risks tends to change over time based on the various issues they are dealing with in their day-to-day lives, as well as the different risk-related behaviour they engage in. Do you feel your HIV risk behaviour has changed during the past month? Six months? Year? Tell me about that.	• *For example, have you felt like your own risk of getting HIV has decreased or increased recently? Over the longer term?*<br>• *(**If less likely**) What risk-reduction strategies have you been less likely to do? For example, have you been less likely to use condoms or not lend or borrow syringes?*<br>• *(**If less likely**) Why have you been less likely to enact these risk reduction strategies?*<br>• *(**If less likely**) Could you describe to me a situation in which you've been less likely to enact risk reduction in the past six months?*<br>• *(**If less likely**) Have any other factors influenced your engagement in these risk behaviours, such as your access to housing or social pressures?*<br>• *(**If no change in risk behaviours**) Why have you continued to enact these risk-reduction strategies over time?*<br>• *(**If no change in risk behaviours**) For example, do you feel that people who inject drugs continue to be vulnerable to HIV transmission, even if they are using various risk-reduction strategies? Why?*<br>• *(**If change in risk behaviours**) Had there been any recent changes in your life? For example:*<br>• *Did you move or become homeless?*<br>• *Did your drug use patterns change?*

	Were you arrested or incarcerated? *Access to health or other support services?* *Had any of your relationships changed significantly?* *Were you feeling estranged from your friends, family, or community?* *How do you feel you were impacted by these changes?* *Do you feel that improvements in HIV treatment mean that you are less vulnerable to HIV transmission? Why?*
(**If applicable**) Do you think that being Indigenous impacts your risk of HIV transmission?	*Why or why not?* *Do you feel your access to harm reduction supplies is impacted? Why or why not?* *Do you feel that your access to health services is impacted? Why or why not?* *Do you feel like you are treated differently by people?* *How does being treated differently by health care providers impact your risk of HIV?* *How does being treated differently by other people impact your risk of HIV?*
III. VACCINE HISTORY AND KNOWLEDGE	
Can you tell me what a vaccine is?<br><br>Read to participant when they've answered: A vaccine is a product that produces immunity from a disease, meaning it prevents disease, but does not cure it. The kinds of vaccines that we often get through needles are an injection of a killed or weakened disease that produce immunity by preparing the immune system to fight that disease in the future.	*What does a vaccine do to the body?* *What makes a vaccine effective or not?*
Were you vaccinated as a child?	*(If yes) Where did you get vaccinated as a child? In school? In a clinic?* *What did you think about vaccines when you were a child? For example, were you scared?* *Do you feel that the vaccines you received as a child were necessary to prevent disease? Why or why not?* *Do you feel that the vaccines you received as a child protect you for the rest of your life? Why or why not?* *(If no) How long do you think vaccines protect people for?* *Do you think it's important for children to get vaccinated? Why or why not?*
(**If applicable**) Are your children vaccinated? Why or why not?	*How did you reach that decision?* *Who did you talk with about vaccinating your children?* *Where were your children vaccinated?* *(If yes) Were your children vaccinated on an alternative schedule? Why?*

Do you think it's important for adults to get vaccinated? Why or why not?<br><br>We need vaccines throughout our lived. Adults need to keep their vaccinations up to date because immunity from childhood vaccines can wear off over time, and adults are at risk for different diseases. Have you received any vaccinations as an adult?	• *(If yes) Which vaccines have you received as an adult?*<br>• *(If yes) When was your most recent vaccination, not including the flu shot? Where did you get it?*<br>• *(If yes) Where do you go for vaccines?*<br>• *(If yes) How many vaccine providers do you estimate you've seen as an adult?*<br>• *(If no) Why have you chosen to not get any vaccinations as an adult?*<br>• *How do you feel about vaccines as an adult?*
Do you get the flu shot every year?	• *Why or why not?*<br>• *(If no) Have you ever had the flu shot?*<br>• *(If no) Why did you stop getting the flu shot?*<br>• *(If never had flu shot) Why have you chosen to never get the flu shot?*<br>• *Do you think you will get the flu shot in the future? Why or why not?*
Can you tell me what you know about herd immunity?<br><br><u>Read to participant:</u> Herd immunity is a kind of protection against a disease that occurs when a large portion of a population has become immune to a disease. If a greater number of individuals are immune, then the chances of someone who is not immune becoming infected are lowered.	• *Have you ever heard of heard immunity before?*<br>• *What have you heard about herd immunity?*<br>• *Do you think it's important to get a vaccine to protect those that cannot get vaccinated?*<br>• *Do you feel that if you are vaccinated, others are protected as well?*<br>• *(After seeing diagram) What do you think about this?*
Do you feel that some vaccines are more important than others?	• *Why or why not?*<br>• *(If yes) Which vaccines do you think are the most important? The lease important? Why do you feel this way?*
**IV. VACCINE HESITANCY**	
Do you believe vaccines are safe for yourself?	• *Why or why not?*<br>• *Are there some vaccines you think are safer than others? Which ones? Why?*<br>• *(If yes) Is there any situation in which you feel vaccines are not safe?*

Have you ever refused a vaccine for any reason?	• (<u>If yes</u>) *Why did you refuse this vaccine?*<br>• (<u>If fear</u>) *Why are you afraid of vaccines?*<br>• *Do you feel it was necessary for you to get this vaccine?*
Have you ever been prevented from getting a vaccine that you intended to get?	• (<u>If yes</u>) *What happened?*<br>• *Were you unable to get the vaccine because of:*<br>o *Distance to the clinic?*<br>o *Time needed to get the clinic?*<br>o *Costs in getting to a clinic?*<br>o *Waiting times?*<br>• *How did this impact your willingness to get any future vaccines?*<br>• *What is the maximum amount of time you would be willing to wait to get a vaccine?*<br>• *Do you feel that your living situation has ever prevented you from getting a vaccination? Why or why not?*
Do you remember anything ever happening in the past that would discourage you from getting vaccines in the future?	• (<u>If yes</u>) *Can you tell me about what happened?*<br>• (<u>If yes</u>) *How did this affect your trust in vaccination?*<br>• *Have you or someone you know ever had a bad reaction to a vaccine that made you reconsider getting vaccines? What happened?*<br>• *Do you believe reports from parents claiming to have lost a child to a disease that could have been prevented by a vaccine? Why or why not?*
When thinking about vaccines, who do you trust the most for information?<br><br>Who do you trust the least?	• *Why do you feel this way?*<br>• *What have you heard about vaccines from (<u>most trusted source</u>)?*<br>• *What have you heard about vaccines from (<u>least trusted source</u>)?*<br>• *Do you feel like you can talk to your health care provider about vaccines? Why or why not?*<br>• *Do you trust your health care provider to safely administer the vaccine to you? Why or why not?*<br>• *Do you ever talk to people in your social circle about vaccination? What do you tell them?*
Have you ever heard anything, positive or negative, about vaccines from the media?	• *What have you heard?*<br>• *What do you think about this?*<br>• *Have reports you heard from the media ever influenced your attitudes towards vaccines? Why or why not?*
Do you think it is possible to get too many vaccines at one time?	• *Why or why not?*<br>• *Do you think vaccines overload the immune system? Why or why not?*<br>• *Are there other (better) ways to prevent diseases than with vaccines? If so, what are they?*
	• *Why do you feel this way?*

How concerned are you that a vaccine might not prevent a disease?	• *Are there some vaccines you're more concerned about than others? Which ones? Why?*<br>• *How concerned are you that a vaccine might causes the disease it was supposed to prevent?*<br>• *How serious do you think vaccine-preventable diseases are? Why?*
**CONSPIRACIES**	
Do you trust that the government is making decisions in your best interest with respect to what vaccines are provided?	• *Why or why not?*<br>• *Do you ever disagree with the vaccines recommended by the government? Why or why not?*<br>• *Do you feel that you are given the best vaccine available by your health care provider or the government?*<br>• *Have you ever felt pushed into receiving a vaccination you didn't want to? What happened?*
Do you feel the people who make vaccines are interested in your health?	• *Why or why not?*<br>• *Do you trust pharmaceutical companies to provide safe and effective vaccines? Why or why not?*
Do you believe reports from parents claiming that their child suffered negative health effects because of a vaccine?	• *Why or why not?*<br>• *What kinds of negative health effects have your heard about being caused by vaccines?*<br>• *Have you ever heard of a vaccine causing negative health effects in the child of anyone you know?*<br>• *(If yes) How did you know the person?*<br>• *(If yes) What did they say happened?*<br>• *(If yes) What do you think about this?*
Do you think the ingredients in vaccines are safe?	• *Why or why not?*<br>• *What dangerous ingredients have your heard of?*<br>• *Why do you think this ingredient is dangerous?*<br>• *Where did you hear about this ingredient?*
What are some other myths you've heard about vaccines that we haven't yet addressed?	• *Where did you hear this?*<br>• *What do you think about the truth of this?*
**V. PERCEPTIONS OF A POTENTIAL HIV VACCINE**	
Have you heard about HIV vaccines before?	• *Can you tell me what you know about HIV vaccines?*<br>• *Do you think this currently exists?*<br>• *If yes, who currently has access to it?*<br>• *If yes, what do you think is the reason it's not available to the public?*<br>• *If yes, when do you think it will be available to the public?*
• Read to participant: A HIV vaccine would protect people who do not have HIV from contracting the virus better than other preventative measures. As of now, there is no HIV vaccine, but many research projects are showing that it may be possible to create an effective HIV vaccine, and there are some clinical trials happening, or about to happen.	
• **ACCEPTABILITY AND FEASABILITY**	

What do you think about a vaccine as an HIV prevention strategy?	• *Do you think an HIV vaccine would be safe? Why or why not?*<br>• *Do you think an HIV vaccine would be effective at preventing HIV transmission? Why or why not?*<br>• *Would you want to receive a vaccine against HIV? Why or why not?*<br>• *(<u>If wants</u>) Would you want to receive the vaccine as soon as it was available? Why or why not?*<br>• *(<u>If doesn't want, or doesn't want right away</u>) Would you want to see what other people who get an HIV vaccine think of it before deciding to get it yourself? Why or why not?*<br>• *How would other people's experience with an HIV vaccine influence your decision to get it or not get it?*<br>• *How would you feel if someone you knew decided to get an HIV vaccine?*<br>• *(<u>If in favour of vaccine</u>) How would other people's vaccination status impact you? For example, would you be worried about other people not being vaccinated? Why or why not?*
How do you think an HIV vaccine might change the way you think about your HIV risk?	• *How would an HIV vaccine impact you engaging in risky behaviours?*<br>• *How do you think an HIV vaccine would influence your decisions around drug use? Sex?*<br>• *Would being vaccinated against HIV make you more or less likely to talk with potential sex partners or the people you use drugs with? Why or why not?*<br>• *Some people think that being vaccinated against HIV might lead people to engage in more risky behaviours. What do you think about this?*
What kind of information would you need to decide whether or not to receive an HIV vaccine?	• *What kind of information would you want about:*<br>o *Safety?*<br>o *Side effects?*<br>o *Pain?*<br>o *Where to get it?*<br>o *Who provides the vaccine?*<br>o *Who makes the vaccine?*<br>• *Where would you prefer to receive this information from? For example, do you think a doctor should provide information about HIV vaccines? A nurse? A pamphlet? The radio?*
Any vaccine can cause side effects, and some vaccines can cause more serious side effects. For example, the vaccine for yellow fever can cause fever, soreness, redness, and swelling in the arm it was given in. How would potential side effects influence your decision to receive an HIV vaccine?	• *Are there any potential side effects that would cause you to refuse an HIV vaccine? If so, what are some examples?*

	• *Would the benefit of receiving an HIV vaccine outweigh the risks of potential side effects for you? Explain.*
**IMPLEMENTATION**	
The next several questions are about implementing an HIV vaccination program…	
How would you prefer to receive an HIV vaccine?	• *Where would you prefer to receive an HIV vaccine? At a health clinic? In the community? Door-to-door? Why?*<br>• *What do you think are the benefits to receiving an HIV vaccine in a clinic? In the community?*<br>• *What do you think are the drawbacks to receiving an HIV vaccine in a clinic? In the community?*<br>• *Who would you prefer to receive an HIV vaccine from? A doctor? A nurse? Why?*<br>• *What do you think are the benefits to receiving an HIV vaccine from a doctor? A nurse?*<br>• *What do you think are the drawbacks to receiving an HIV vaccine from a doctor? From a nurse?*<br>• *Do you think it's more important for people to receive medical consultation before getting an HIV vaccine, or for it to be convenient? Explain?*<br>• *Would you feel comfortable asking your doctor for an HIV vaccination? Why or why not?*
Some vaccinations are divided into multiple doses and administered over a period of time. What do you think about this in relation to an HIV vaccine?	• *Would you want to receive an HIV vaccine if you had to get more than one dose? Why or why not?*<br>• *How many doses do you think is reasonable?*<br>• *How much time would you be willing to wait between doses?*<br>• *How do you think requiring multiple doses would impact people's willingness to receive an HIV vaccine?*
Who do you think would get an HIV vaccine?	• *Are there any groups of people you feel are more likely to want an HIV vaccine than others? Who?*<br>• *Who do you think it is most important to vaccinate against HIV? Why?*<br>• *Who do you think an HIV vaccine program would target first? Why?*<br>• *Do you think it's fair to prioritize certain groups receiving an HIV vaccine before others? Why or why not?*<br>• *How would you feel if your doctor recommended you get an HIV vaccine because you are thought ot be "high risk"?*
Not all vaccines are publically funded, and some vaccines are only available for free for some groups of people. What are your thoughts on this?	• *Are there certain groups of people who you think an HIV vaccine should be free for? Which groups? Why?*<br>• *Do you think an HIV vaccine should be publically funded for everyone? Why or why not?*<br>• *Would you be willing to pay out of pocket for an HIV vaccine? Why or why not?*<br>• *(If yes) what do you think is a fair cost?*

SECTION THREE: PERSPECTIVES ON PreP	
**Introduction:** Another risk-reduction strategy that is becoming available in some settings is **Pre-exposure prophylaxis (or PrEP)**. Within this approach to HIV prevention, people who are **not** HIV positive can take medication and reduce their likelihood of getting HIV. It requires the HIV negative person to take a pill once a day, including before and after the risk exposure. And, it is very important that the pill is taken each day, or it becomes less and less effective as doses are missed. At this point, PrEP is not freely available in BC – but, there are ongoing discussions by the province to potentially do so in the near future. Other settings have begun to make it available. We want to hear your thoughts on PrEP.	• *Have you heard about PrEP before today? Tell me what you knew about it. Where did you hear about it?*<br>• *What do you think about PrEP as an HIV prevention strategy?*<br>• *How would you feel if someone you knew decided to take PrEP?*<br>• *How would you feel if your doctor offered you PrEP, or suggested you started taking PrEP?*<br>• *Have you been in a situation or can you imagine a situation where accessing PrEP would have been helpful? Can you tell me about that?*<br>• *Would you feel comfortable asking your doctor for PrEP? Under what circumstances?*<br>• *How do you think PrEP might change the way you think about HIV risk and having sex? For example, would it make you more or less concerned about your partners' potential HIV status? Would you be more or less likely to engage in conversations about HIV and risk with your sex partners? Why or why not?*<br>• *How would PrEP influence your decisions around sharing syringes with others? For example, would you be more or less likely to share syringes? Why?*<br>• *Would being on PrEP make you more or less likely to talk about the risk of HIV transmission with the people you share drugs with? Why?*
**Regular engagement with care:** For those who decide to take PrEP, it is important that they do so through a health care provider. For example, those who get PrEP are usually required to test for HIV at a regular interval of every three months.	• *What are your thoughts on the idea of taking PrEP and getting tested for HIV every three months? How might that work in your day-to-day life?*<br>• *Is regular HIV testing something you would be willing to do in order to get PrEP? Where do you know you can go to get tested?*<br>• *In considering your own experiences, would it be possible for you to make sure you show up for regular appointments (e.g., every three months) with your doctor so you could get PrEP?*<br>• *For example, how would this look in your current/previous circumstances?*<br>• *What would you need in your life in order to make sure you were able to meet with your doctor regularly – if you had to – to be on PrEP?*
**Intermittent/"on-demand" PrEP**: Using PrEP "on demand" is also effective at preventing the	• *Could you see yourself planning in advance to take PrEP (e.g., before having sex with someone)?*

sexual transmission of HIV. This means that, prior to having sex, two pills are taken two and 24 hours before sex, and then one pill 24 and 48 hours later. How does this version of "on demand" PrEP make you think differently (or not) about PrEP?	• *Do you feel that you would be able to plan in advance of a sexual experience in this kind of a way? Tell me about how this sort of an experience might take place, given your current situation (e.g., where you're living, how you plan your sexual activities).*
**Long-Acting PrEP:** Scientists are currently working on improved versions of PrEP that could come in a variety of different forms. For example, future forms of PrEP could be longer acting (e.g., it could last up to a many weeks or months).	• *How does a longer-lasting kind of PrEP influence whether or not this is something you would consider taking?*<br>• *Would you be more or less likely to consider using PrEP if it could last for a "longer" time? Why?*<br>• *How long would you want PrEP to last if you were going to use it as a way to prevent getting HIV? Tell me about that.*
**Long-Acting Injectable PrEP:** Future versions of PrEP might also come in the form of an injectable, rather than a pill. It is possible that this would be a longer-acting form of PrEP, and that it might act for weeks or even months before the next dose is needed. What are your thoughts on taking a form of PrEP that is injectable?	• *Do you think an injectable form of PrEP would be something you might benefit from? Tell me about that.*<br>• *Would you be more or less likely to consider using PrEP if it could be given to you in the form of an injection (e.g., rather than a pill)? Why?*<br>• *What would be the ideal way – for you – of taking PrEP to prevent HIV? Why?*
**Injection-related risks and PrEP:** Some settings are recommending that people who use various forms of drugs (e.g., injection drugs) take PrEP to reduce the risk of HIV acquisitions.	• *Do you think PrEP is something that you would like to take to prevent yourself from getting HIV through injection drug use? Why?*<br>• *Some people have told us that PrEP would lead people to engage in more risky behaviour, as they would feel "bullet proof". What are your thoughts on that?*<br>• *What types of risks do you feel that people would take if they were on PrEP? What are your thoughts on that?*<br>• *What, if any, risks do you feel that you would be more likely to take if you were on PrEP?*<br>• *How would you think about someone who injects drugs and who is on PrEP? Why?*
**Sexual-related risks and PrEP:** Some groups are viewed as being 'higher risk' for sexual transmission of HIV. For example, gay and bisexual and other men who have sex with men are usually considered to be at a higher risk. As a result, doctors may recommend young MSM engage in various risk-reduction practices (e.g., regular condom use). Do you feel like you are somebody who should be offered PrEP to prevent the sexual transmission of HIV?	• *What are your thoughts on being offered PrEP if you are thought to be "high-risk" by your health care provider because you are involved with sex work or identify as gay, bisexual or other MSM?*<br>• *Which groups do you think should be 'targeted' to go on PrEP to prevent sexual transmission of HIV? Tell me why you think this.*<br>• *Some people have told us that it is not fair to target specific groups of people for these kinds of HIV interventions, as this could be based on hurtful assumptions and/or stereotypes. What do you think about this?*

HIV PREVENTION POLICY/CAMPAIGNS	
Have you ever seen or heard of any vaccination campaigns?	• *(If yes) Can you tell me about the campaigns you've seen?*
	• *(If yes) What was memorable about this to you?*
	• *(If yes) What information about the vaccine do you remember being included in the campaign?*
	• *(If yes) Do you feel you learned enough about the vaccine being advertised to make a decision on whether or not to get it? Why or why not?*
	• *(If not enough info) What more information do you feel you needed?*
	• *In general, do you feel you get enough information about vaccines and their safety?*
If you were to plan a campaign for an HIV vaccine, what would it look like?	• *What age do you feel should be targeted?*
	• *Which groups of people should be targeted?*
	• *Where do you think would be the most effective place for an HIV vaccine campaign?*
	• *Who do you think would be the most difficult to engage with?*
	• *What information should be included in a public HIV vaccination campaign?*
	• *What information should not be included?*
	• *What information should be given only by a medical professional if someone is interested in an HIV vaccine?*
Who do you think would be the most responsive to an HIV campaign?	• *Why do you feel this way?*
	• *What is it about this group that would make them want to get an HIV vaccine?*
	• *Do you feel people who are most at risk would be most likely to respond to an HIV vaccination campaign?*
	• *How would people's perception of their own risk of HIV infection impact their responsiveness to a vaccination campaign?*
What do you think about compulsory vaccination? For example, some schools don't allow children to attend who haven't been vaccinated. What do you think about this?	• *Do you think an HIV vaccine should be mandatory? Why or why not?*
	• *(If yes) Who should an HIV vaccine be mandatory for? Why?*
	• *(If no) Is there anyone you think should be encouraged to get an HIV vaccine?*
	• *Is there an age group that should be given an HIV vaccine, similar to HPV vaccinations given to women born in certain years?*
	• *How would you feel if it was mandatory that you receive an HIV vaccine?*
	• *How would you feel if you had to report your HIV vaccination status?*

If you were to plan a campaign for PreP, what would it look like?	• *What age do you feel should be targeted?*
	• *Which groups of people should be targeted?*
	• *Where do you think would be the most effective place for a PreP campaign?*
	• *Who do you think would be the most difficult to engage with?*
	• *What information should be included in a public PreP vaccination campaign?*
	• *What information should not be included?*
	• *What information should be given by a medical professional if someone is interested in PreP?*

V. WRAP UP QUESTIONS

"Thank you for sharing your experiences with us? We have a couple of final questions about your views on stimulant treatments…"

- Is there anything we haven't discussed about HIV vaccines or other methods of HIV prevention that you feel is important for us to know?
